# Ribosylation Rapidly Induces α-Synuclein to Form Highly Cytotoxic Molten Globules of Advanced Glycation End Products

**DOI:** 10.1371/journal.pone.0009052

**Published:** 2010-02-04

**Authors:** Lan Chen, Yan Wei, Xueqing Wang, Rongqiao He

**Affiliations:** 1 State Key Laboratory of Brain and Cognitive Sciences, Institute of Biophysics, Chinese Academy of Sciences, Beijing, China; 2 Laboratory of Mental Health, Institute of Psychology, Chinese Academy of Sciences, Beijing, China; 3 Graduate University of Chinese Academy of Sciences, Beijing, China; Mayo Clinic, United States of America

## Abstract

**Background:**

Alpha synuclein (α-Syn) is the main component of Lewy bodies which are associated with several neurodegenerative diseases such as Parkinson's disease. While the glycation with *D*-glucose that results in α-Syn misfold and aggregation has been studied, the effects of glycation with *D*-ribose on α-Syn have not been investigated.

**Methodology/Principal Findings:**

Here, we show that ribosylation induces α-Syn misfolding and generates advanced glycation end products (AGEs) which form protein molten globules with high cytotoxcity. Results from native- and SDS-PAGE showed that *D*-ribose reacted rapidly with α-Syn, leading to dimerization and polymerization. Trypsin digestion and sequencing analysis revealed that during ribosylation the lysinyl residues (K_58_, K_60_, K_80_, K_96_, K_97_ and K_102_) in the C-terminal region reacted more quickly with *D*-ribose than those of the N-terminal region. Using Western blotting, AGEs resulting from the glycation of α-Syn were observed within 24 h in the presence of *D*-ribose, but were not observed in the presence of *D*-glucose. Changes in fluorescence at 410 nm demonstrated again that AGEs were formed during early ribosylation. Changes in the secondary structure of ribosylated α-Syn were not clearly detected by CD spectrometry in studies on protein conformation. However, intrinsic fluorescence at 310 nm decreased markedly in the presence of *D*-ribose. Observations with atomic force microscopy showed that the surface morphology of glycated α-Syn looked like globular aggregates. thioflavin T (ThT) fluorescence increased during α-Syn incubation regardless of ribosylation. As incubation time increased, ribosylation of α-Syn resulted in a blue-shift (∼100 nm) in the fluorescence of ANS. The light scattering intensity of ribosylated α-Syn was not markedly different from native α-Syn, suggesting that ribosylated α-Syn is present as molten protein globules. Ribosylated products had a high cytotoxicity to SH-SY5Y cells, leading to LDH release and increase in the levels of reactive oxygen species (ROS).

**Conclusions/Significance:**

α-Syn is rapidly glycated in the presence of *D*-ribose generating molten globule-like aggregations which cause cell oxidative stress and result in high cytotoxicity.

## Introduction

Alpha-synuclein (α-Syn), a protein located in the cytoplasm which contains 140 amino acids, is a member of a highly conserved family of proteins consisting of α-, β-, and γ-synuclein. α-Syn, an abundant neuronal protein involved in synaptic function, has become extremely important in studies on the pathology of several common neurodegenerative diseases, including Parkinson's disease (PD), dementia characterized by the presence of Lewy bodies (LB), and multiple system atrophy [1], [2], [3], [4], [5]. The prominence of α-Syn in LB in the substantia nigra is one of the cardinal pathological features of PD [6]. PD is a chronic, progressive movement disorder, with four primary symptoms: tremor, bradykinesia, rigidity and postural instability. After Alzheimer's disease, PD is the most common neurodegenerative disorder, with an estimated lifetime risk of approximately 1 in 100 persons [7]. While the native protein is naturally unfolded under physiological conditions, the α-Syn protein in Lewy bodies from brain tissues of PD patients is misfolded and becomes protease-resistant [8]. Many research groups have found that chemical modifications such as glycation [9], sumoylation [10], and phosphorylation [11] are involved in α-Syn misfolding and aggregation.

In recent years the glycation of α-Syn in Lewy bodies has become one of important problems in this field. Many authors have suggested that glycation of α-Syn is a pathological hallmark of LB in PD patients [9], [12], [13]. Advanced glycation end-products (AGEs) and α-Syn are co-localized in the brain of the patients at both the early and advanced stages of PD [9]. The intracellular accumulation of AGEs precedes α-Syn-positive inclusion body formation, and extracellular AGEs accelerate the process of intracellular α-Syn-positive inclusion body formation [12].

So far, although glycation of α-Syn has received considerable attention, the role of *D*-ribose in glycation has not been studied. *D*-ribose is a naturally occurring pentose monosaccharide present in all living cells (also in blood) and is an essential component for biological energy production. Seuffer has determined the concentration of *D*-ribose (0.01–0.1 mM) present in cerebrospinal fluid (CSF) [14]. It is used to synthesize nucleotides, nucleic acids, glycogen, and other important metabolic products. *D*-ribose is also formed in the body from conversion of *D*-glucose via the pentose phosphate pathway. Thus, *D*-ribose is present both intracellularly and extracellularly, and has opportunities to react with proteins and produce glycated derivatives. For this reason, glycation of α-Syn protein with *D*-ribose needs to be investigated.

Glycation affects the conformation and function of proteins such as hemoglobin [15] and albumin [16], [17], leading to a marked loss of their functions [18]. Abnormal modification, including glycation, induces neuronal proteins to misfold and form amyloid fibrils in a stepwise process from prefibrils to fibrils [19]. More research is needed to elucidate abnormal modifications such as glycation which play an important role in the aggregation and toxicity of α-Syn [6]. The significance of α-Syn fibrillization in PD is supported by a growing body of evidence. Insoluble α-Syn is the major component of Lewy bodies, cytoplasmic intraneuronal inclusions that are the defining neuropathological feature of PD [1], [20].

Dobson and colleagues report that only globular, pre-fibrillar aggregates display cytotoxicity, whereas mature fibrils are substantially harmless [21], [22]. Globule-like protein aggregations (pro-amyloid fibrils) are highly toxic to neurons [21], [23], [24], [25], [26]. Recently, formation of molten globule-like states has been reported during prolonged glycation of human serum albumin [27]. However, there are no reports in the literature that glycation with *D*-ribose induces protein misfolding into molten globules during aggregation. The characteristics and cytotoxicity of molten globule-like protein states induced by either *D*-ribose or *D*-glucose have not been clarified.

In this laboratory, we have observed that glycation induces inactivation and conformational change in *D*-glyceraldehyde-3-phosphate dehydrogenase [28], [29]. While *in vitro* glycation of α-Syn with *D*-glucose does not result in distinct conformational changes of the protein [30], ribosylation of bovine serum albumin (BSA) and neuronal Tau protein leads to ThT-positive aggregations with high cytotoxicity [31], [32]. We have also found that Tau protein aggregates in the presence of formaldehyde at low concentrations and forms ThT-positive deposits which are cytotoxic [33]. Here, we investigate the sequential order of ribosylation of lysinyl residues of α-Syn and whether it generates cytotoxic molten globule-like aggregations.

## Results

### Ribosylation of Alpha-Synuclein

Recombinant human α-synuclein was expressed in *E. coli* cells and purified by a stepwise procedure as described in the Materials and Methods. The α-Syn protein ran as a single band on 15% SDS-PAGE with a purity of ∼95% (Fig. S1). The purified α-Syn protein was confirmed by Western blotting with a monoclonal antibody (data not shown).

**Figure 1:**
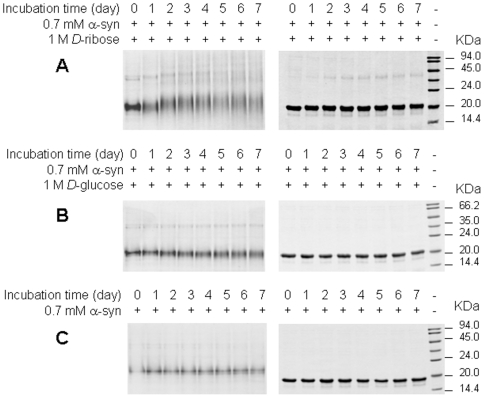
Ribosylated α-Syn on native-PAGE and SDS-PAGE. 0.7 mM α-Syn was incubated with 1 M *D*-ribose (A) and 1 M *D*-glucose (B) for 0–7 days, and then loaded on 15% native-PAGE (left) and 15% SDS-PAGE (right) gels. (C) Native α-Syn alone was used as a control.

During ribosylation, we incubated α-Syn with *D*-ribose and took aliquots at different time intervals for electrophoretic SDS-PAGE and native PAGE (Fig. 1). Protein dimerization began around day 3 after the start of incubation (Fig. 1A). However, dimerization of α-Syn was not detected in the presence of *D*-glucose (Fig. 1B) or in the absence of sugar (Fig. 1C). Analysis using mass spectrometry showed that the molecular mass of glycated α-Syn (incubated for 7 days) increased to ∼16,297 Da, about 1,848 Da higher than that for native α-Syn, indicating that, on average about 14 ribosyl groups (132 Da each) were bound to α-Syn.

**Figure 2:**
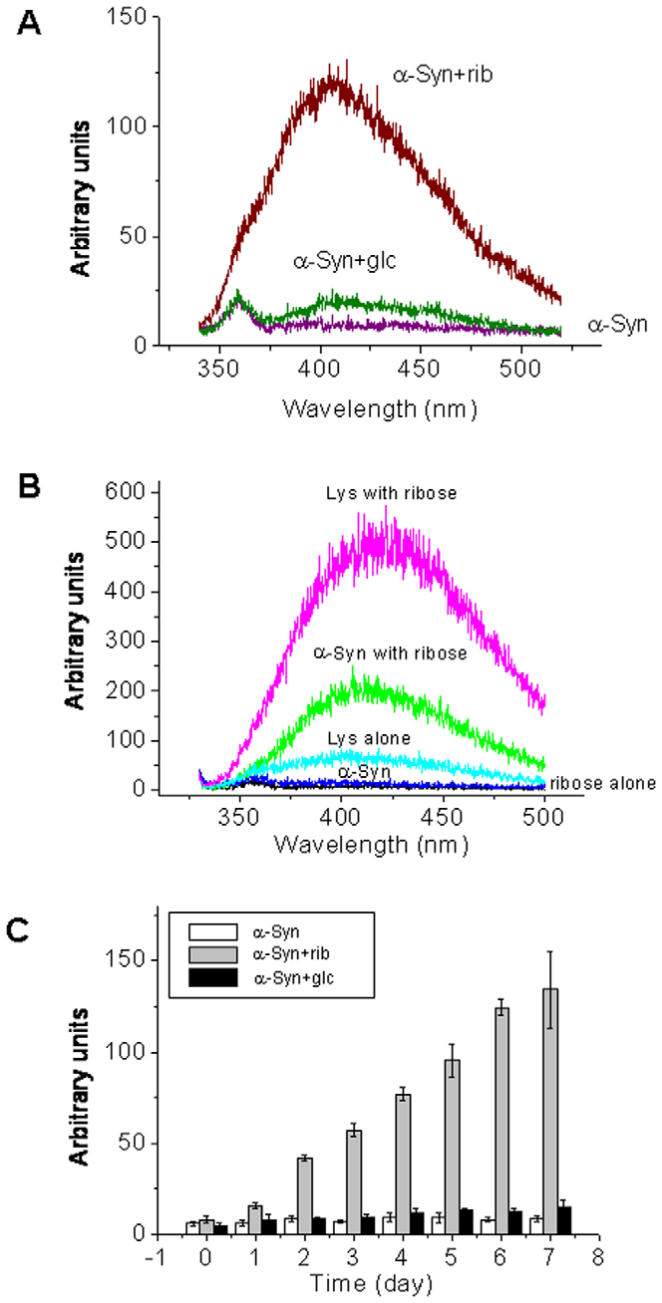
Changes in fluorescence during protein riboslyation. Glycation conditions were the same as those in Fig. 1. (A) Aliquots were taken for measurements of the intrinsic fluorescence at an excitation wavelength of 320 nm after incubation for 3 days. (B) Characteristics of the fluorescence of glycated products. (C) Changes in the maximal fluorescent intensity (λ_ex_320 nm; λ_em_410 nm) were monitored while α-Syn was incubated with *D*-ribose, or glucose for different time intervals.

According to Liu and colleagues [34], glycation of a protein produces a new fluorescence derivative (λ_ex_320 nm, λ_em_410 nm), and thus fluorescence is commonly used to monitor the formation of AGEs. As shown in Fig. 2A, the fluorescence emission intensity of α-Syn incubated with *D*-ribose (α-Syn + rib) for 7 days was much stronger than for that incubated with *D*-glucose (α-Syn + glc). α-Syn alone, used as a negative control, showed no fluorescence at 410 nm. To characterize whether the fluorescence at 410 nm resulted from the reaction of *D*-ribose with lysinyl residues, we resuspended lysine in a *D*-ribose solution under the experimental conditions, and observed a strong fluorescent emission at 425 nm by excitation at 320 nm (Fig. 2B). Native α-Syn, lysine and *D*-ribose did not show marked fluorescence. This suggests that the fluorescent derivative is resulted from the reaction of *D*-ribose with ε-amino groups of the lysinyl residues of the protein.

**Figure 3:**
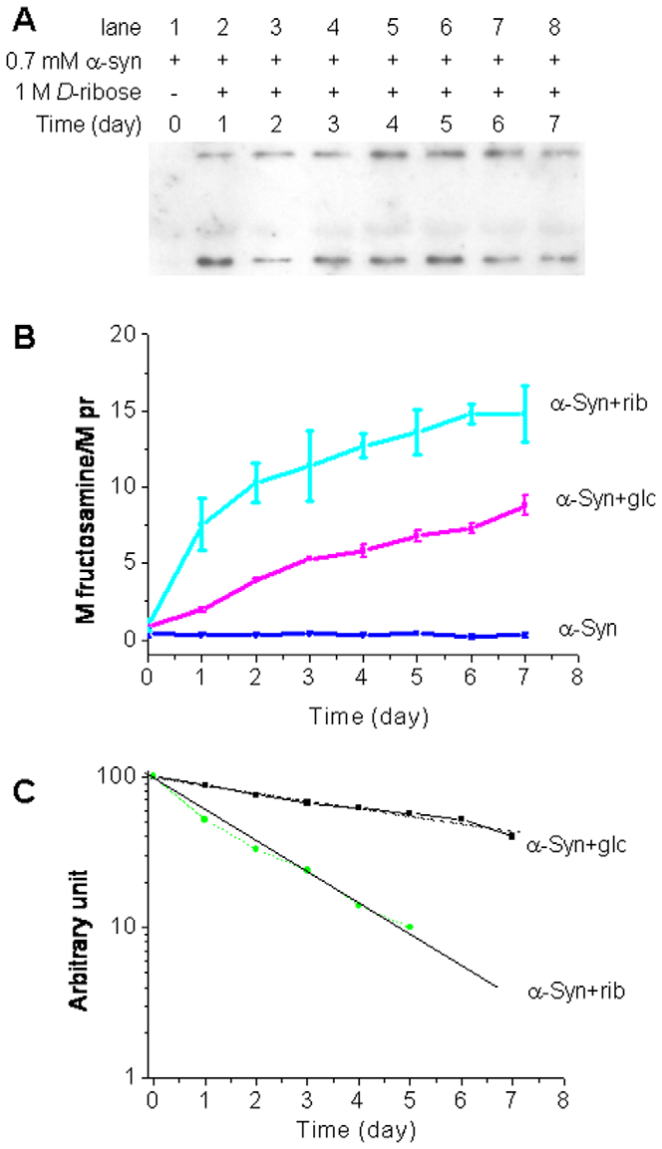
Detection of AGEs by Western blotting and NBT assays. The glycation conditions of α-Syn were the same as those in Fig. 1. (A) Western blotting of the aliquots (ribosylated α-Syn) with anti-AGEs was performed at different time intervals. (B) Fructosamine was assayed with NBT. (C) Data in panel B were analyzed according to Tsou's method [74].

To confirm whether AGEs were formed during glycation with *D*-ribose, a monoclonal antibody that is commonly used to assay AGEs (6D12) was used in Western blotting [35]. The monoclonal antibody recognized both monomers and dimers of the glycated protein from day 1 to day 7 of the incubation. α-Syn in the presence of *D*-glucose or absence of sugar did not show significant spots on the membrane under the experimental conditions (not shown). Thus our results suggest that AGEs resulting from ribosylated α-Syn start to form at the initial stage of the incubation (Fig. 3A).

### Kinetics of Ribosylation

Changes in the fluorescence at 410 nm at different time intervals (days) were measured. The emission intensity of ribosylated α-Syn increased markedly with incubation time (Fig. 2C). But glucosylated and native α-Syn did not show marked increases in the fluorescence under the experimental conditions used in this study. This demonstrates that glycation of α-Syn with *D*-ribose occurs much more rapidly than that with glucose.

Fructosamine is a common product of glycation and the fructosamine content of a given protein reflects its degree of glycation [36], [37]. The assay of fructosamine with nitroblue tetrazolium (NBT) is based on a color change correlated with the reduction of NBT to monoformazan by Amadori rearrangement products in an alkaline buffer. Here, we used NBT to monitor the time course of formation of fructosamine during glycation. As shown in Fig. 3B, the absorbance at 540 nm increased markedly with time. The absorption value increased rapidly on day 1 and then gradually reached a plateau. Analysis of the yield of fructosamine on day 7 of the incubation showed that about 15 moles of *D*-ribosyl groups and 8 moles of *D*-glucosyl groups were coupled with one mole of α-Syn.

Kinetic studies showed that the increase in the absorbance at 540 nm (formation of fructosamine) underwent a monophasic time course with a first order rate constant of 5.79×10^−6^⋅s^−1^ for ribosylated α-Syn, while the first order rate constant was 1.33×10^−6^⋅s^-1^ for glucosylated α-Syn (Table 1). This suggests that ribosylation of α-Syn occurs much faster than glucosylation.

**Table 1:** First order rate constants for changes in fructosamine and fluorescence during the glycation of α-Syn protein in the presence of *D*-ribose or *D*-glucose.

Kinetic measurements		Relaxation time (h)	First phase	Second phase
Grey density	Monomer Oligomer	– 24	– 5.67	– 1.48
Fructosamine (α-Syn+rib)		–	5.79	–
Fructosamine (α-Syn+glc)		–	1.33	–
Fluorescence at 310 nm		–	19.04	7.11
Fluorescence at 410 nm		24	3.03	16.70
ThT fluorescence(α-Syn)		24	1.90	7.01
ThT fluorescence(α-Syn+rib)		–	2.13	7.01

Rate constants are in 10^−6^.

**Figure 4:**
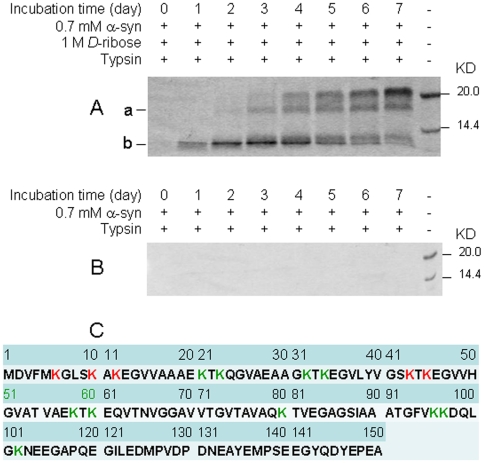
Digestion of glycated α-Syn in the presence of trypsin. (A) Aliquots were digested by trypsin at a mass ratio [α-Syn]/[trypsin] of 20∶1 (37°C, 1 h). (B) Native α-Syn alone was used as a control. (C) α-Syn amino acid sequence. The protein fragments ‘a’ and ‘b’ as shown in panel A were sequenced as described [75]. Lysine residues are shown in green, except those at the cleavage sites (as determined by sequencing) which are shown in red.

In order to observe the time course of ribosylation of lysinyl residures, we used trypsin (which specifically recognizes lysinyl residues) to digest ribosylated α-Syn (Fig. 4A) Ribosylated α-Syn became resistant to trypsin digestion (mass ratio of ribosylated α-Syn to protease: 20∶1). On day 1 of the incubation, one protein band (apparent molecular mass ∼12.8 KDa) appeared on SDS-PAGE gels, followed by another digested band (apparent molecular mass ∼18.4 KDa) on day 3. The density of the ribosylated α-Syn band became thicker as incubation time increased. In contrast, native α-Syn was rapidly degraded under the experimental conditions used (Fig. 4B). Ribosylation blocked the cleavage sites which trypsin recognized. According to sequence analysis, trypsin cleaved at K_43_-T_44_ and K_45_-E_46_ released the ∼12.8 KDa fragment, and that cleaved at K_6_-G_7_, K_10_-A_11_ and K_12_-E_13_ produced the ∼18.4 KDa fragment detected by SDS-PAGE (Fig. 4C and Fig. S2). This indicates that the α-amino groups of the lysinyl residues (K_58_, K_60_, K_80_, K_96_, K_97_ and K_102_) in the C-terminal region were more rapidly ribosylated than those (K_6_, K_10_, K_12_, K_21_, K_23_, K_32_, K_34_, K_43_ and K_45_) in the N-terminal region.

### Conformational Changes of Ribosylated Alpha-Syn

**Figure 5:**
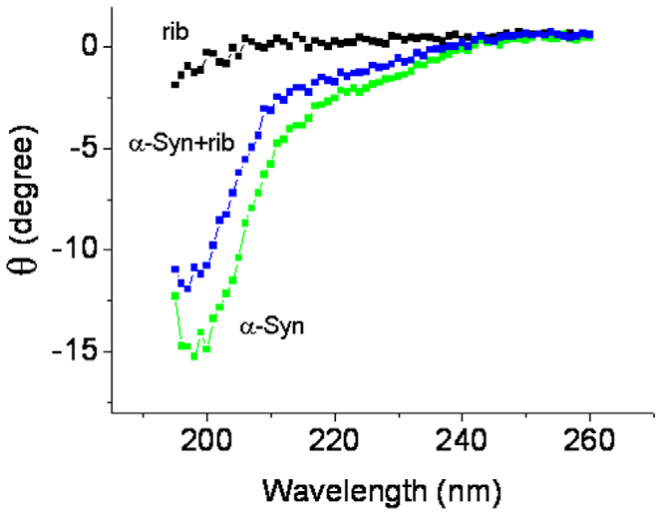
Changes in the CD spectra of α-Syn in the presence of *D*-ribose. A representative CD spectra of α-Syn incubated with *D*-ribose (day 3) is shown. Conditions for glycation of α-Syn with *D*-ribose were the same as those in Fig. 1.

Although human α-Syn is known to be a natively unfolded protein [38], we wanted to determine whether the secondary structure of α-Syn changes during ribosylation. As shown in Fig. 5, α-Syn has a far-UV CD spectrum typical of an unfolded polypeptide chain. The spectrum of ribosylated α-Syn was almost the same as that of native α-Syn indicating that the secondary structures of ribosylated α-Syn do not undergo significant changes. Similar to native α-Syn, the modified protein lacked ordered secondary structures under the experimental conditions used.

**Figure 6:**
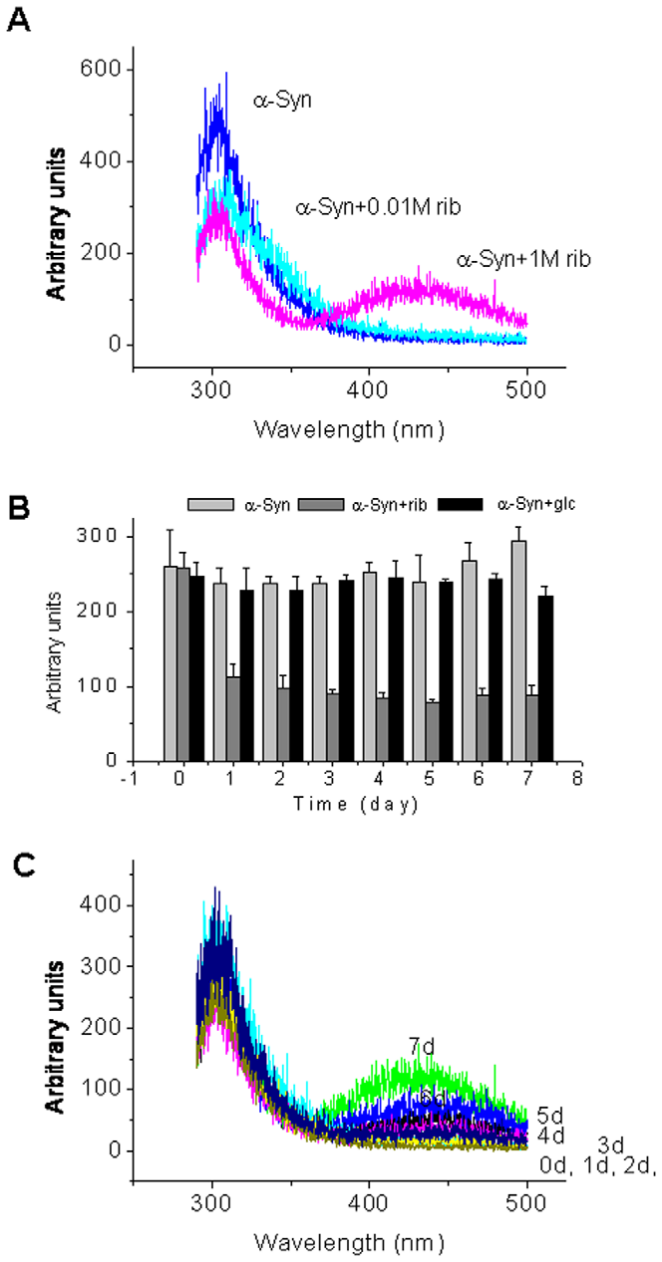
Conformational changes in glycated α-Syn observed by intrinsic fluorescence. Experimental conditions were the same as those in Fig. 1. (A) Fluorescence spectra of α-Syn glycated with *D*-ribose at an excitation wavelength of 280 nm. (B) The kinetics of fluorescence in *D*-ribose glycated with α-Syn was analyzed at different time intervals (λ_ex_ 280 nm; λ_em_ 310 nm). (C) The incubation time-dependent increase in non tryptophan fluorescence is shown for 0 to 7 days.

To investigate whether ribosylation induced a change in the tertiary structure of α-Syn, we took aliquots at different incubation time intervals and measured the protein intrinsic fluorescence (at 310 nm, arising from tyrosinyl residues). As shown in Fig. 6A, the intensity of intrinsic fluorescence decreased when α-Syn was incubated with *D*-ribose at different concentrations. In the time course study (Fig. 6B), the emission intensity of the protein intrinsic fluorescence decreased with time, especially on day 1 of the incubation. Under the experimental conditions used here, changes in the intrinsic fluorescence of α-Syn incubated with *D*-glucose were not marked, as was the case for native α-Syn in the absence of sugar. It appears that there may be changes in the tertiary structure of α-Syn during ribosylation.

In kinetic studies, changes in the intensity of intrinsic fluorescence decreased and underwent a biphasic procedure: a fast and slow phase (Table 1). The first order rate constant of the fast phase was 19.04×10^−6^⋅s^−1^, showing a rapid change in the tertiary structure at the initial stage of ribosylation, followed by a relatively slow change.

Fig. 6C shows us an interesting result. Fluorescence at around 440 nm appeared with ribosylated α-Syn with an excitation wavelength of 280 nm (the excitation wavelength for detecting the intrinsic fluorescence of tyrosinyl residues). The emission intensity increased during the glycation process, accompanied with a blue shift from 450 nm to 440 nm, indicating that an energy transfer occurred between the tyrosinyl residues and the ribosylated groups, and confirming that the ribosylated groups are spatially close to the tyrosinyl residues within the protein.

**Figure 7:**
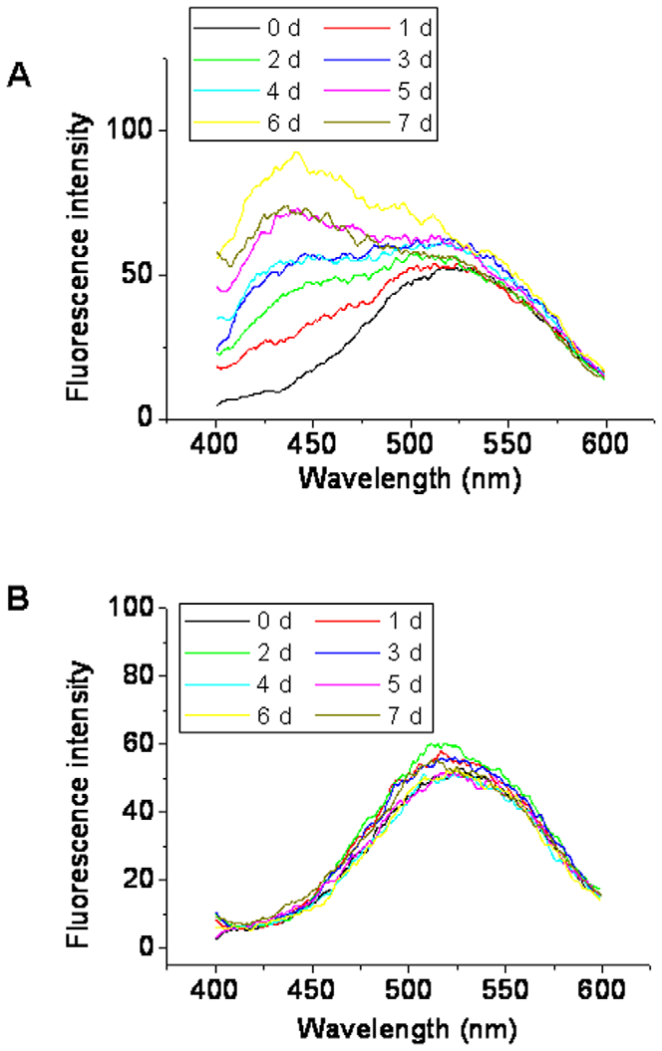
Changes in ANS fluorescence of α-Syn during glycation. Experimental conditions were the same as those in Fig. 1. (A) ANS (70 µM) was added to samples of ribosylated α-Syn at different time intervals. Fluorescence spectra of ANS were recorded at λex 350 nm. (B) Native α-Syn alone was used as a control.

The fluorescent molecule 8-anilino-1-naphthalenesulfonate (ANS), which is frequently used to demonstrate the presence of partially folded conformations of globular proteins [39], was used to clarify whether any hydrophobic patches become exposed to the exterior of the α-Syn molecule. Fig. 7A shows that ribosylation increased the ANS fluorescence intensity (525 nm) and induced a blue shift (∼100 nm) of the ANS λ_max_. Under the same conditions, little blue shift was observed with native α-Syn in the absence of *D*-ribose (Fig. 7B). These results show that α-Syn is converted into a partially folded conformation with solvent-exposed hydrophobic patches present within protein molten globules during ribosylation.

### Ribosylated α-Syn in Molten Globules

**Figure 8:**
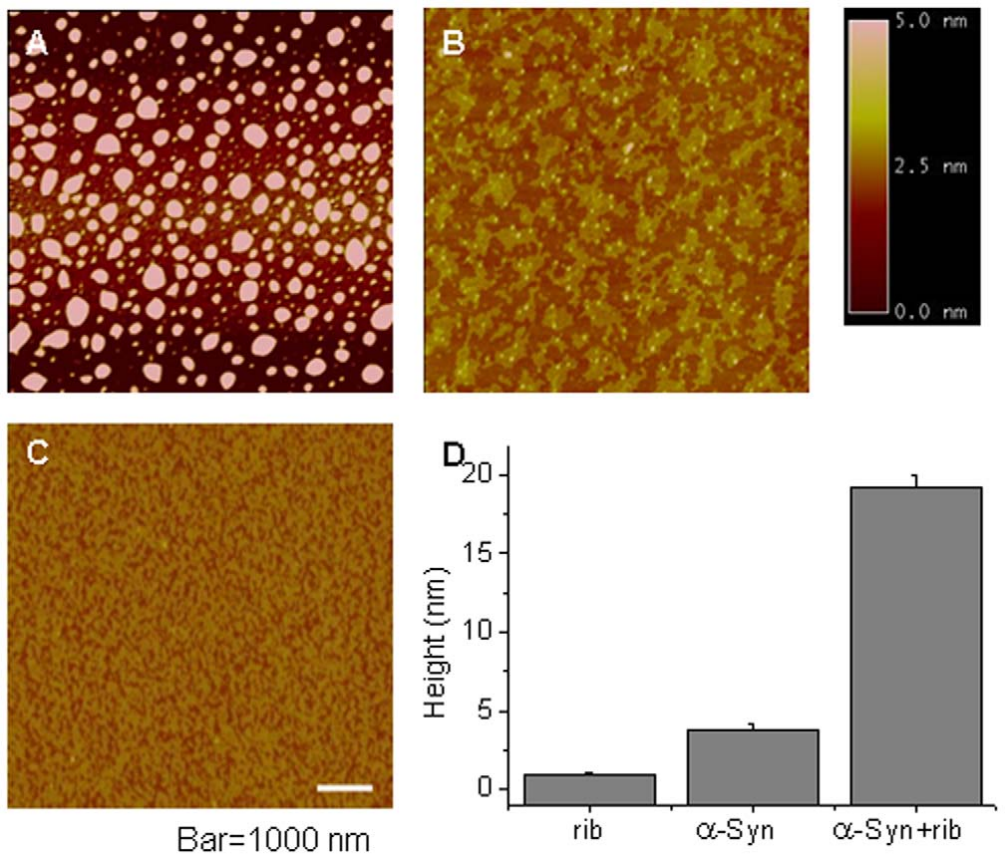
Glycated α-Syn deposits were imaged by atomic force microscopy. (A) Aliquots of α-Syn incubated with *D*-ribose for three days were taken for observation by AFM. (B) Native α-Syn was employed as a control. (C) *D*-Ribose alone was used as a negative control. (D) Horizontal diameter at mid-height.

To investigate the occurrence of protein molten globules, atomic force microscopy (AFM) was employed to observe glycated α-Syn. As shown in Fig. 8A, globule-like aggregates of ribosylated α-Syn (3 days) were numerous under AFM using the tapping model in air. The average height of glycated α-Syn was 19.22±0.71 nm. Globular aggregates were not present in control samples of native α-Syn, however some surface irregularities on the mica surface with a height of 3.77±0.38 nm were observed (Fig. 8B, D). Globule-like forms were not observed when *D*-ribose alone was employed as a control (Fig. 8C). This suggests that ribosylated α-Syn is polymerized and exists in molten globules.

**Figure 9:**
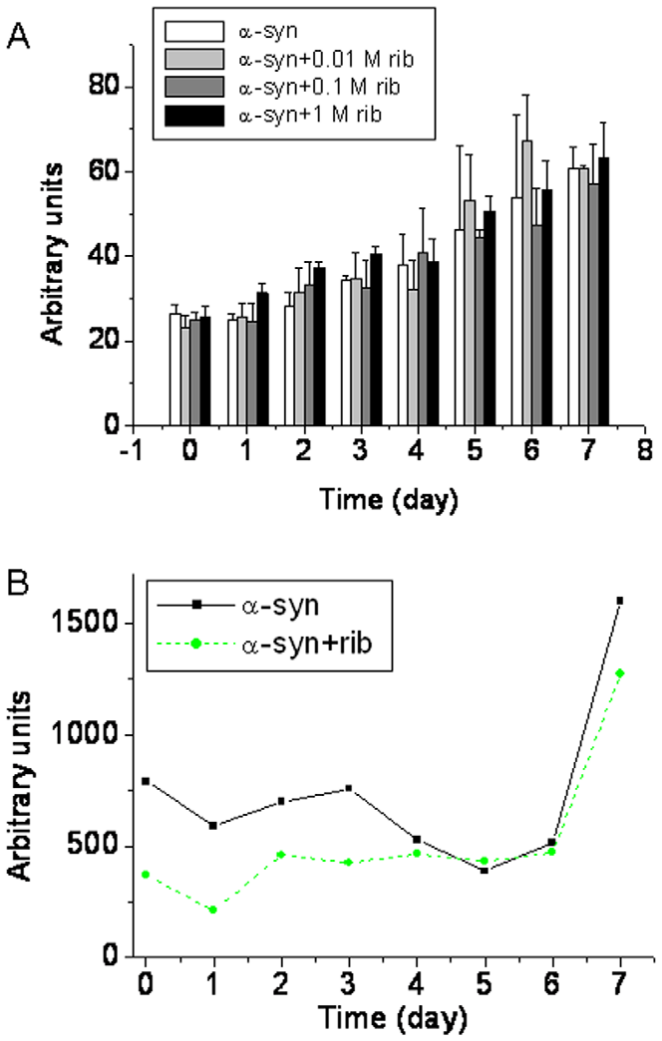
Changes in the fluorescence of thioflavin T and light scattering in the presence of ribosylated α-Syn at different time intervals. (A) ThT (30 µM) was added to samples of α-Syn in different concentrations of *D*-ribose for different time intervals. The intensity of ThT fluorescence was recorded (λ_ex_ 450 nm; λ_em_ 485 nm). The kinetics of the increase in the fluorescence emission of ThT with glycated α-Syn is shown. (B) Aliquots were taken for measurements of the intensity of scattered light (λ_ex_ 480 nm; λ_em_ 480 nm) under the same conditions. Native α-Syn alone was used as a control.

The fluorescence emission of thioflavin T, a specific fluorescent marker for intermolecular β-sheets (amyloid-like aggregates), is often used to clarify the characterization of protein aggregates [40], [41]. Here we employed ThT to investigate the intermolecular β-sheets in ribosylated α-Syn polymers [42]. ThT fluorescence (λ_ex_ 450 nm, λ_em_ 485 nm) was slightly increased during the ribosylation of α-Syn as shown in Fig. 9A. However, the intensities of the ThT fluorescence of ribosylated α-Syn were similar to those of native α-Syn incubated in the absence of *D*-ribose. Furthermore, the emission intensities of the protein in the presence of *D*-ribose at different concentrations were similar to those of the control. The positive ThT emission which is characteristic of aggregation further suggests that ribosylated α-Syn molecules exist in molten globules.

To further confirm that ribosylated α-Syn exists as molten globules, light-scattering of the sample solutions was measured (Fig. 9B). Light-scattering intensities (480 nm) did not show a significant change between ribosylated and native α-Syn until day 7 of the incubation, indicating that both were soluble in solution, although glycation induced protein dimerization and polymerization. This further suggests that the dimers and polymers of ribosylated α-Syn were in molten globular states under the experimental conditions employed.

### Molten Globules of Ribosylated α-Synuclein Have High Cytotoxicity

**Figure 10:**
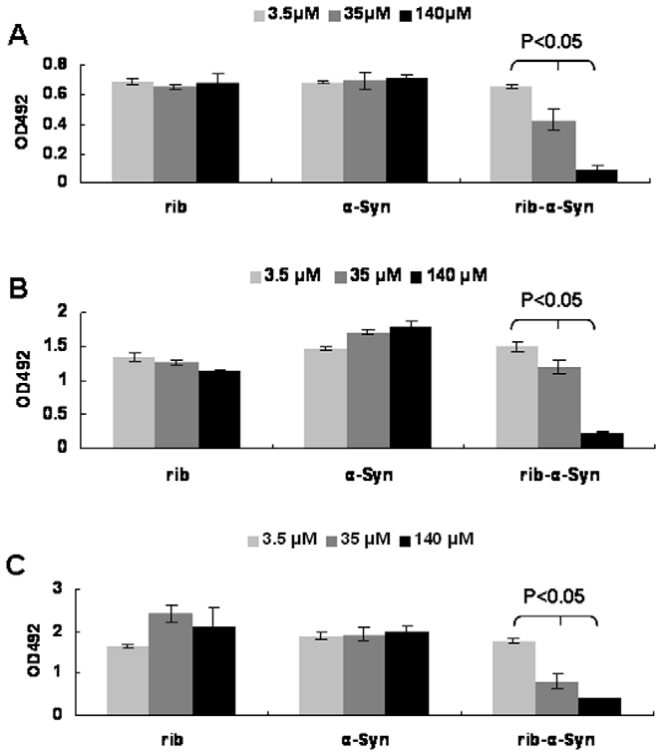
Cell viability was measured with the MTT assay. Cell viability of SHSY5Y cells was measured after adding 3.5 µM, 35 µM and 140 µM ribosylated α-Syn at 24 h (A), 48 h (B) and 72 h (C).

Since ribosylated α-Syn is present as molten globules, its cytotoxicity is a matter of great concern. The effect of ribosylated α-Syn on the viability of SH-SY5Y cells was examined using MTT assays (Fig. 10). The number of viable cells decreased significantly when they were incubated with ribosylated α-Syn for 8 h, while cell viability did not significantly change in the presence of native α-Syn or *D*-ribose. The reduction in cell viability induced by ribosylated α-Syn was affected in a concentration dependent manner at different time intervals (24, 48 and 72 h).

**Figure 11:**
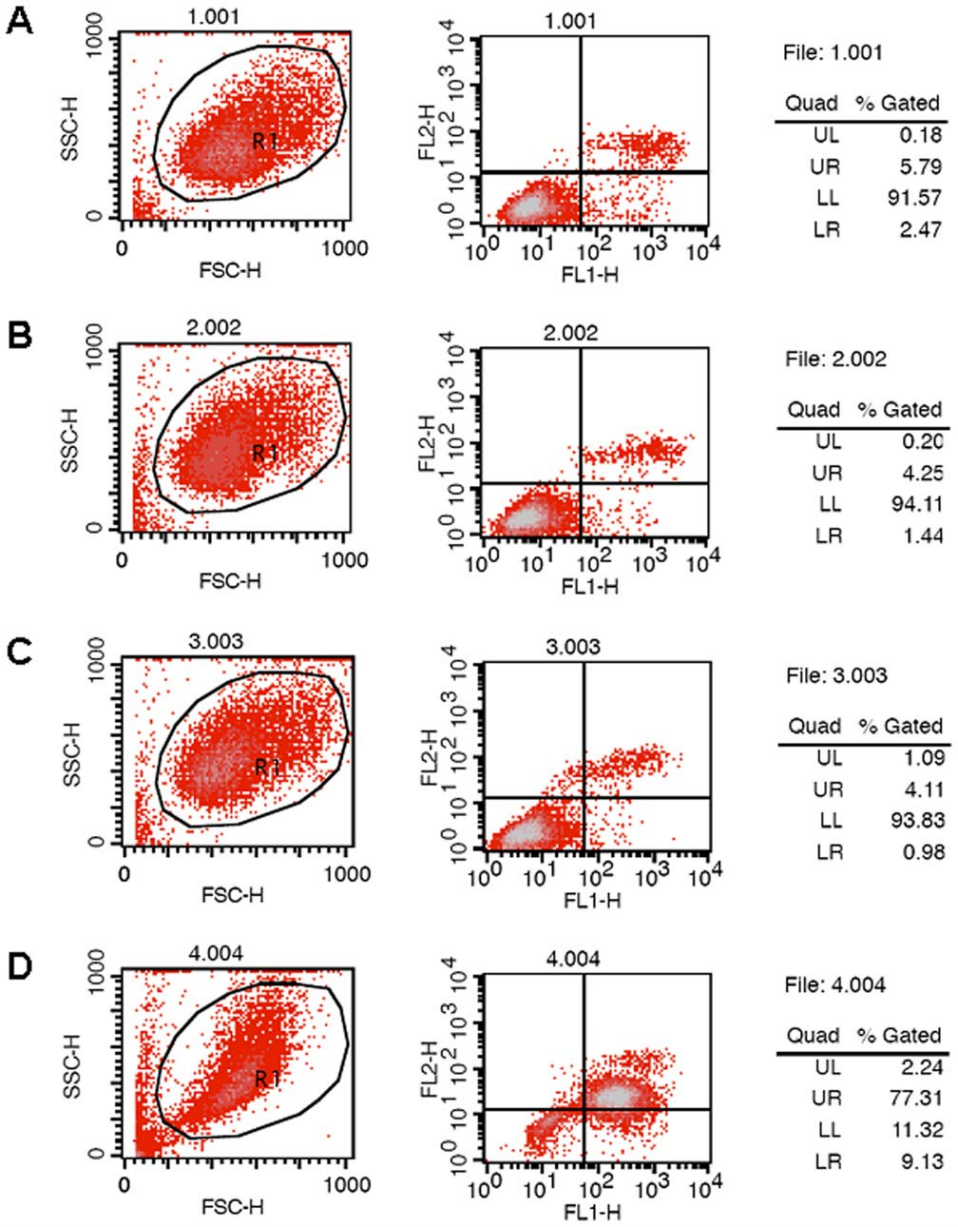
Ribosylated α-Syn induces SH-SY5Y cell apoptosis. SH-SY5Y cells were treated with ribosylated α-Syn for 8 h and then cultured for 24 h, before analysis by flow cytometry (D). Normal cells were used as controls (A), cells incubated with *D*-ribose (B) and α-Syn (C) are shown.

In order to reveal the cause of the reduction in cell viability induced by glycated α-Syn, annexin V/PI assays were examined on a flow cytometer (Fig. 11). After incubation with ribosylated α-Syn for 8 h, a significant increase in early apoptosis (LR, 9.13%) and late apoptosis/necrosis (UR, 77.31%) rates were observed (Fig. 11D), while the ratios were lower than 3% (LR) and 6% (UR) in the presence of native α-Syn, *D*-ribose and untreated control cells (Fig. 11C, B, A). These results indicate that ribosylated α-Syn induces cell apoptosis and necrosis.

**Figure 12:**
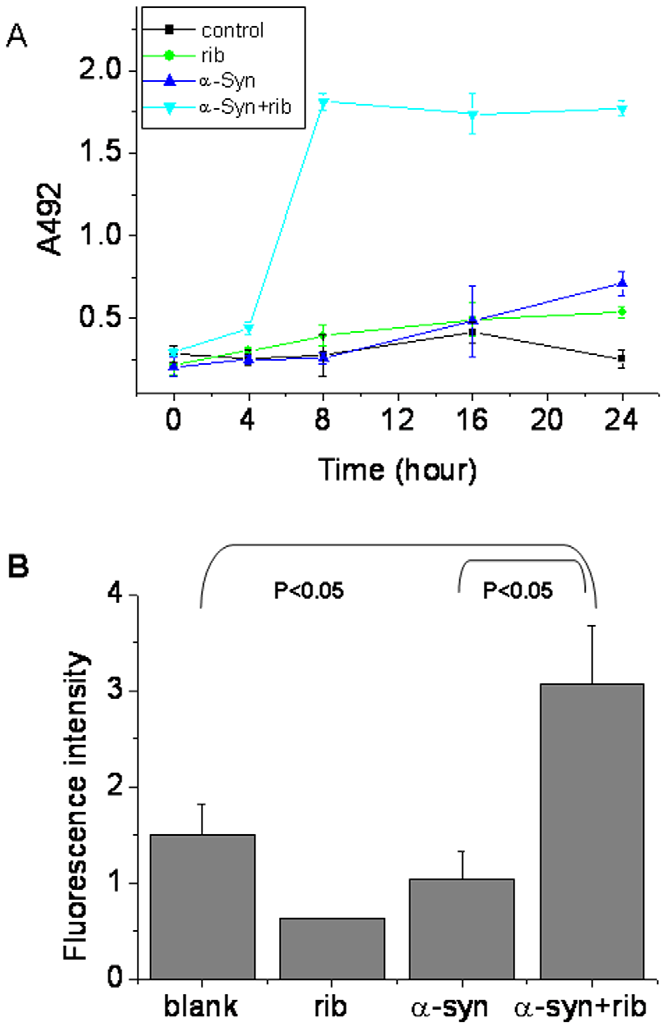
LDH assay and measurement of reactive oxygen species in SH-SY5Y cells. (A) LDH activity was measured using a cytotoxicity detection kit (Roche, Switzerland). (B) The levels of reactive oxygen species in cells treated with glycated α-Syn for 24 h were measured. Cells treated with α-Syn, *D*-ribose and a blank were used as controls.

To confirm that cells are damaged in the presence of ribosylated α-Syn, release of LDH from the SHSY5Y cells was measured (Fig. 12A). Glycated α-Syn at high concentration (35 µM) was able to trigger cell death in 8 h of incubation, however, native α-Syn or *D*-ribose alone did not show significant cytotoxicity at the same concentrations. These results reveal that molten globules of ribosylated α-Syn have high cytotoxicity and induce apoptosis and necrosis of cells.

More interestingly, in order to determine whether free radicals are involved in SHSY5Y lesions, the level of intracellular reactive oxygen species (ROS) was measured (Fig. 12B). After SHSY5Y cells were incubated with ribosylated α-Syn for 8 h, the level of ROS increased significantly in comparison to cells which were incubated with native α-Syn or *D*-ribose alone. This suggests that the increased level of intracellular ROS is involved in the cytotoxicity.

## Discussion

α-synuclein is vulnerable to aggregation under pathological conditions and forms insoluble fibrils that are a characteristic of Lewy bodies, a pathological hallmark of the neurodegenerative diseases. According to recent studies [21], [43], globule-like protein aggregations are significantly toxic to neural cells. In addition to posttranslational modifications such as phosphorylation [44], and sumoylation [13], glycation is commonly regarded as a cause of disrupted protein function and produces cytotoxic products such as AGEs, leading to medical complications [45], [46]. Our results have shown that α-Syn protein is rapidly glycated and induced to form globule-like aggregations in the presence of *D*-ribose. Here we have investigated whether ribosylation of α-Syn yields protein molten globules with ThT-positive characteristics and high cytotoxicity to SHSY5Y cells.

### High Efficiency in Ribosylation of α-Syn

As is the case with glucosylation, ribosylation occurs between the amino groups of a protein, particularly between the epsilon amino groups of lysinyl residues. Such carbohydrate-amino acid reactions, often termed as “maillard” or “non-enzymatic browning” reactions, result in linkages that are not hydrolyzed by digestive enzymes although the amino acids may still be recovered from the protein by acid hydrolysis [47]. Histidinyl and tryptophanyl residues also react in this way but do so much more slowly with reducing sugars. The argininyl residue is also able to react with *D*-ribose, but α-Syn does not contain this residue.

As an efficient glycating agent, *D*-ribose is a naturally occurring pentose monosaccharide and an essential component for energy production in the human body. *D*-ribose is present in all living cells and blood, and is present in the human brain [48] as well as in CSF [14]. Moreover, α-Syn has an unstable conformation with a flexible peptidyl chain. Weinreb and colleagues showed that the α-Syn protein exists in a native “unfolded” conformation, having an irregular structures [49]. Most lysinyl residues are probably exposed to the exterior of the α-Syn molecule because of the hydrophilic nature of the ε-amino group. The “worm-like” conformation of the α-Syn protein, whose lysinyl residues are exposed, creates more opportunities for reactions with reducing sugars than does the conformation of globular proteins, explaining why α-Syn is rapidly glycated in the presence of *D*-ribose.

**Figure 13:**
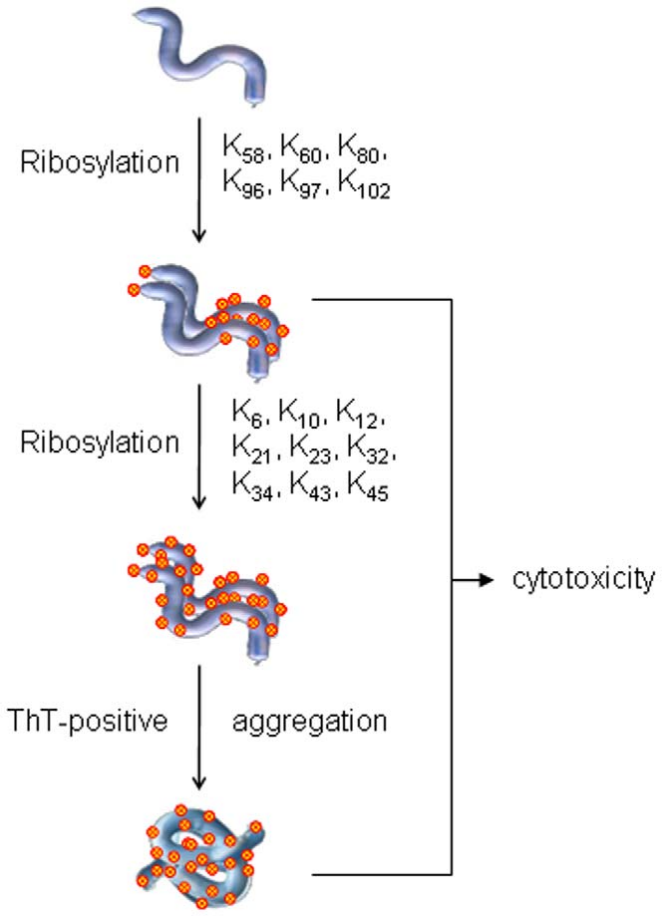
A putative scheme for the aggregation of ribosylated α-Syn protein into highly cytotoxic molten globules.

Our mass spectrometry results suggest that approximately 14 *D*-ribosyl groups bind to one molecule of α-Syn protein. Results from NBT assays described above are not as precise as that of mass spectrometry. As α-Syn contains 15 lysinyl residues, we suggest that most of the lysinyl residues are available and can react with *D*-ribose. As shown in Fig. 4, *D*-ribose reacts first with the ε-amino groups of lysinyl residues in the C-terminal region (K_58_, K_60_, K_80_, K_96_, K_97_, K_102_ and α-amino group), and then with the K_6_, K_10_, K_12_, K_21_, K_23_, K_32_, K_34_, K_43_ and K_45_ groups of the N-terminal region. A possible order for ribosylation of the α-Syn protein is shown in Fig. 13. That is, ribosylation of α-Syn undergoes a rapid modification in the C-terminal lysinly residues and a slow modification in the N-terminal region, accompanied with protein aggregation.

### D-Ribose and Glucose in Glycation of α-Syn

So far, however, most research groups have been studying glycation with *D-*glucose rather than *D*-ribose even though *D*-ribose is present both intracellularly and extracellularly. One hand, according to, e.g. Mak et al. [50], the CSF glucose concentration is about 4 mM, i. e. 40–400 times higher than the ribose concentrations. Thus, in brain glucose appears to be much more abundant than ribose. On the other hand, *D*-glucose has a much lower reducing potential than that of *D*-ribose. We have previously shown that glycation of α-Syn with *D*-glucose is a slow procedure, and glucosylated α-Syn had no significant cytotoxicity under the experimental conditions [30]. Glycation of BSA with *D*-ribose is much faster than that with *D*-glucose [31]. Even though the concentration of D-ribose is 10 times lower than that of glucose, ribosylation is still much faster that glucosylation. This is due to the low glycation efficiency of *D*-glucose compared with *D*-ribose. However, it is still unclear whether *D*-glucose is the most important sugar in α-Syn glycation *in vivo* although AGEs and α-Syn have been found in Lewy bodies. Whether the sugar on the glycated α-Syn is ribose or not has not been confirmed in the literature. Although it is shown here that ribose reacts faster with alpha-synuclein in vitro, glucose that might be quantitatively more relevant in vivo for AGE formation and alpha-synuclein pathogenesis should be further investigated.

We postulate that ribosylation of α-Syn is more efficient than glucosylation based on the following observations: (1) The formation of 410 nm fluorescence in ribosylation was faster than that in glucosylation as shown in Fig. 2A; (2) the NBT assay showed that glycation of α-Syn with *D*-ribose was faster and produced more fructosamine than with *D*-glucose (Fig. 3B); (3) The first order rate constant for changes in the intrinsic fluorescence of α-Syn in the presence of *D*-ribose was greater than that in the presence of *D*-glucose as shown in our previous work [30]; and finally, (4) the cytotoxicity of ribosylated α-Syn (20 µM in MTT and LDH assays) is distinctly stronger than for glucosylated α-Syn (data not shown).

### Ribosylation of α-Syn Promotes Molten Globules

According to previous reports, it is still unclear whether glycation with *D*-glucose can induce α-Syn protein to form amyloid aggregates. Here, however, we have shown that *D*-ribose reacted quickly with α-Syn to promote aggregation of glycated products into ThT-positive aggregates (Fig. 9A). Fibrils were not observed under the experimental conditions. In particular, the first order rate constants showed that the formation of 410 nm fluorescence was rapid from day 5 of glycation, and paralleled a decrease in the intrinsic fluorescence of the α-Syn protein. Under AFM, the ribosylated aggregates looked like globules on the mica surface. Fibrils of ribosylated α-Syn could not be observed on the mica diaphragm under our experimental conditions (Fig. 8A). Thus glycation with *D*-ribose induces the α-Syn protein to form globular aggregations.

Molten globules of a protein are one of the early intermediate states of protein misfolding [51], [52]. We conclude that ribosylated α-Syn is present as molten globules based on these observations. (1) As mentioned above, the far-UV CD spectra (Fig. 5) showed little change in the secondary structures of the protein. Similar results have been observed during glycation of albumin, in which the far-UV CD of the glycated protein was not significantly changed compared with that of the native albumin used as a control [17]. (2) The intrinsic fluorescence of α-Syn with *D*-ribose changed markedly during the incubation period, suggesting a distinct conformational change in the tertiary structure [53], [54]. (3) The positive results of the ANS assay reveal the exposure of some hydrophobic patches of the ribosylated α-Syn protein molecules, another piece of evidence indicating the molten globule state [55]. (4) We have not observed the formation of amyloid-fibrils with ribosylated α-Syn under the experimental conditions used. (5) We have observed that ribosylated α-Syn is present as globular-like aggregations on the mica diaphragm using AFM. (6) The intensity of the light scattering of ribosylated α-Syn samples did not markedly increase until day 7 of the incubation, suggesting that the glycated protein is soluble in solution, namely that it is present as molten globules.

### α-Syn Molten-Globules Are Characteristic of AGEs

Glycation is a non-enzymatic post-translational modification that involves a covalent linkage between a sugar and an amino group of a protein forming a ketoamine [56]. Subsequent oxidation, fragmentation and/or cross-linking of ketoamines lead to the production of AGEs [57], [58]. AGEs are thought to play an important role in PD by oxidation of α-Syn. The AGE formation has detrimental effects on the structure and function of affected proteins [59]. Accumulation of AGEs has been implicated in normal aging and in the pathogenesis of diabetes-associated complications and PD. In the case of ribosylated α-Syn, the highly cytotoxic glycated products appear to result from globular aggregations of AGEs. Anti-AGE monoclonal antibodies recognized both glycated monomers and glycated dimers; that is, AGEs could be monomers, dimers and polymers of ribosylated α-Syn. Cytotoxicity of ribosylated α-Syn was marked under the experimental conditions employed (Fig. 10 and Fig. 12A), suggesting that molten globules of α-Syn have high cytotoxicity.

Appearance of fluorescence at 410 nm has been observed during the incubation of α-Syn with *D*-ribose (Fig. 2). This fluorescence is thought to be diagnostic for AGEs resulting from glycation [34], [60], [61], [62]. The presence of this fluorescence shows that AGE-dimerization and AGE-polymerization of α-Syn occur, because (1) the ribosylated α-Syn dimer appears in the early stages of glycation as shown by SDS-PAGE (Fig. 1); (2) globular aggregations of ribosylated α-Syn were observed under AFM (Fig. 8); (3) the kinetic increase in the intrinsic fluorescence is a biphasic process (with a fast-slow phase) similar to that of dimerization of the glycated protein; and (4) the increase in the 410 nm fluorescence underwent a slow-fast phase that is similar to that of ThT fluorescence (Table 1).

It is interesting, however, that during glycation a new fluorescence appears at around 450 nm after excitation at 280 nm (Fig. 6C). According to Matiacevich and colleagues [60], this most likely represents the formation of AGEs. Our results showed the energy transfer between the intrinsic fluorophore (tyrosinyl residues) and the glycated fluorescent derivatives. The intrinsic fluorescence of tyrosinyl residues around 310 nm was absorbed by the glycated fluorescent derivatives and emitted at 450 nm. This evidence shows that the glycated fluorescent derivatives are spatially close to the tyrosinyl residues in ribosylated α-Syn dimers and polymers.

### Ribosylation of Proteins Case by Case

In general, ribosylation of different proteins has some common characteristics: formation of globule-like protein aggregations (molten globules), and production of AGEs with high cytotoxicity. However, different proteins undergo different changes in their conformational features. Dimerization of the α-Syn protein clearly occurs as shown by the ribosylation on SDS-PAGE, but polymers are not stable (as shown by native-PAGE, Fig. 1). For neuronal Tau protein, the monomer, oligomer and polymer are present and can be seen to follow a clear ribosylation procedure on SDS-PAGE [32]. For BSA, a glycated monomer is present, while ribosylated dimers and polymers cannot be clearly detected on SDS-PAGE [31]. Thus, although *D*-ribose initially reacts with an amino group of a protein undergoing a universal glycation procedure (Schiff's base reactions between the sugar and the protein, followed by conversion to ketoamines through Amadori rearrangement, and finally formation of AGEs), different proteins show different conformational changes during ribosylation.

### Ribosylated α-Syn Has High Cytotoxicity

In this research we have studied α-Syn glycation in the presence of *D*-glucose. As mentioned above, the cytotoxicity of ribosylated α-Syn was much higher than that of glucosylated α-Syn under our experimental conditions. This is consistent with results obtained previously for the glycation of BSA and Tau protein, an important protein involved in Alzheimer's disease which causes impairments of cells [31], [32].

This work has showed that ribosylated α-Syn is able to significantly inhibit the growth of SHSY5Y cells (Fig. 10). The dose-dependent cytotoxicity observed here reveals that glycated α-Syn may disturb neural cell metabolism and viability. Results from the flow cytometric analysis of annexin V/PI support this viewpoint and show that apoptosis and necrosis of SHSY5Y cells was induced by ribosylated α-Syn. Nie and colleagues have reported that formaldehyde-treated globular aggregates of Tau possess cytotoxicity [33]. Similarly, soluble ThT-positive molten globules of ribosylated α-Syn also have the ability to promote SHSY5Y cell death. Thus, molten globules of ribosylated α-Syn may be useful as a molecular model for simulating protein misfolding *in vitro*. In the light of its high cytotoxicity, it appears that ribosylation may play an important role in α-Syn pathological processes.

AGE-recombinant Tau generates reactive oxygen intermediates and induces oxidative stress when introduced into the cytoplasm of SHSY5Y neuroblastoma cells [56]. In a previous report we have shown that apoptosis induced by ribosylated BSA and Tau is involved in oxidative stress [31]. It appears that the oxidative signaling pathway is at least involved in the cell death induced by the ribosylated α-Syn protein.

### Glycated α-Syn Has Been Found in the Brain Tissues of PD Patients, Especially in the Lewy Bodies

It has been reported that glycated α-Syn is found in the brain of patients with PD [9]. Many research groups have studied the relationship between glycation of α-Syn protein and the formation of LB [12]. Furthermore, glycated α-Syn induces lipid peroxidation *in vivo* and results in lesions within cells [13]. This suggests that glycation may play a role in stabilizing the α-Syn aggregations that are related to LB formation in PD [9]. Although glycated α-Syn protein has been found in LB, it has been difficult to clarify whether the bound sugar is *D*-ribose or *D*-glucose. This question needs further investigation.

Finally, ribosylated α-Syn rapidly generates AGEs in protein molten globules which induce SHSY5Y cell death by cell oxidative stress. These molten globules of ribosylated protein have some similar characteristics to pathological aggregations in their high cytotoxicity and could thus be used as an *in vitro* model for research to identify drugs that are valuable for disease treatment, such as the “anti-glycation” treatment for PD and other neurodegenerative diseases [63].

## Materials and Methods

### Expression and Purification of α-Syn

Human wild type α-Syn was expressed in *Escherichia coli* BL21(DE3) cells using a pRK172-α-Syn plasmid (a kind gift from Dr. Hongyu Hu, Institute of Biochemistry and Cell Biology, Shanghai Institutes for Biological Sciences, China) and purified as described by Huang et al [64]. The resultant α-Syn was present as a single band on SDS-PAGE with a purity of ∼95% as shown in Fig. S1. The purified α-Syn was lyophilized and stored at -70°C before use.

### Preparation of Ribosylated Protein

α-Syn was dissolved in 20 mM Tris-HCl (pH 7.4) to yield a stock solution of 20 mg/ml. This solution was then resuspended with *D*-ribose (Sigma, USA) prepared in Tris-HCl (pH 7.4) to a final concentration of 10 mg/ml α-Syn and 1 M monosaccharide. α-Syn alone and in the presence of *D*-glucose (Sigma, USA) were used as controls. Reaction mixtures were incubated at 37°C for different lengths of time (from 0 to 7 days). All solutions were filtered with 0.22 µm membranes (Millipore, USA).

### NBT Colorimetric Fructosamine Assay

The extent of glycation of individual α-Syn preparations was assessed using the NBT (Ameresco, USA) assay as described previously [17], [37], [65], [66]. 200 µl of 0.75 mM NBT was added to a 96-well microplate along with 10 µl of the sample or standard. The kinetics of the reduction of NBT by the fructosamine group (0.1 M carbonate buffer, pH 10.35) were measured at 540 nm after incubation for 30 min at 37°C using an MK3 microplate reader (Thermo, USA). Standard curves were generated by addition of 10 µl of 1-deoxy-1-morpholino-D-fructose (1-DMF, Sigma-Aldrich, USA). The quantity of fructosamine formed was established by comparing with standard curves (R^2^>0.99).

### SDS-PAGE

Aliquots of glycated protein samples were subjected to SDS-PAGE. For the digestion experiment, α-Syn (35 µM) and trypsin (3.5 µM) were mixed in Tris-HCl buffer (pH 7.4) to give a final volume of 100 µl, and incubated at 37°C for 1 h. Aliquots were subjected to electrophoresis using Bio-Rad (USA) electrophoretic equipment.

### Western Blotting

Aliquots of α-Syn incubated with *D*-ribose for different durations were subjected to electrophoresis. The proteins were then transferred onto PVDF membranes, and probed with anti-AGEs (dilution = 1∶1000, 6D12, Wako, Osaka, Japan) followed by goat anti-mouse horseradish peroxidase (HRP) (KPL, Gaithersburg, Maryland, USA) at a dilution of 1∶2000. Immunoreactive bands were visualized using enhanced chemiluminescence (Pierce, USA).

### Fluorescence Measurements

Intrinsic fluorescence of α-Syn (5 µM) was monitored on an F4500 fluorescence spectrophotometer (Hitachi, Japan). The emission spectrum from 290 nm to 500 nm was recorded by excitation at 280 nm at 25°C. To measure the energy transfer from the intrinsic fluorescence (tyrosinyl residues) to ribosylated fluorescent derivatives, glycated aliquots were excited at 280 nm and the emission spectra from 300 nm to 500 nm was recorded. To assess the fluorescence of AGEs derived from glycated protein, we monitored an emission spectrum from 320 nm to 500 nm (λ_ex_ = 320 nm) as described previously [67].

### Measurement of Thioflavin T Binding Fluorescence

α-Syn (5 µM) and thioflavin T (30 µM, Sigma, USA) were mixed at 25°C, and the fluorescence was subsequently measured (λ_ex_450 nm; λ_em_485 nm) as described [68].

### ANS Binding Assay

A stock solution of 3.3 mM ANS (Sigma, USA**)** was prepared in 20 mM Tris-HCl (pH 7.4). α-Syn (7 µM) and ANS (70 µM, Sigma-Aldrich, USA) were mixed at room temperature for 1 h, and the fluorescence was subsequently measured by recording the emission spectrum from 400 to 600 nm (λ_ex_ = 350 nm).

### Light-Scattering Assay

In light scattering experiments the intensity of scattered light from ribosylated α-Syn (5 µM) was recorded using a F4500 fluorescence spectrofluorometer, whose incident and scattering monochromators were both set at 480 nm. α-Syn protein alone was used as a control.

### Observation with Atomic Force Microscopy

The conditions for producing ribosylated α-Syn were as described above. All solutions used were filtered through a 0.22 µm filter. Samples were diluted to the desired concentration using Tris-HCl buffer (pH 7.4), and aliquots (10 µl) were allowed to adsorb onto the mica and were kept at room temperature for 5 min before observation. Observation under AFM (Mutiplemode-I, Digital Instruments, USA) was carried out as described previously [68].

### Circular Dichroism (CD) Spectropolarimetry Measurements

Far-UV CD measurements were performed on a circular dichroism chiroptical spectrometer (Jasco J-720, Japan). Samples in a 1 mm quartz cuvette were maintained at 25°C with a circulating water bath. The spectra of ribosylated α-Syn (50 µM) were measured (195 nm–260 nm) with a step size of 1.0 nm. Each measurement was repeated 10 times and averaged. The background of the corresponding buffers in the absence of protein and *D*-ribose was subtracted for all the samples.

### Cell Culture in the Presence of Ribosylated α-Syn

SHSY5Y cells were cultured in Dulbecco's modified Eagle's medium (DMEM, Gibco, USA) containing 10% fetal bovine serum (Hyclone, USA), 100 IU/ml penicillin and 100 µg/ml streptomycin (Sigma, USA) at 37°C in a humidified 5% CO_2_ incubator. Cells were grown to 70–80% confluence in 25 mm diameter dishes and subcultured every fourth day. To test the effect of ribosylated α-Syn on cell growth, the culture medium was replaced with serum-free medium before the addition of the glycated protein. The cells were incubated with different concentrations of ribosylated α-Syn (3.5 µM and 35 µM) for 8 h, after which the medium was changed to DMEM with 10% fetal bovine serum for further culture.

### Cell Viability Test

We used a standard 3-(4,5-dimethylthiazol-2-yl)-2,5-diphenyl tetrazolium bromide (MTT, Sigma) test, which was slightly modified from that of Mayo and Stein [69]. SHSY5Y cells were seeded onto a 96-well plate at a concentration of 10^5^ cells per well and either exposed or not exposed to ribosylated α-Syn for 8 h. MTT (0.5 mg/ml) was added after 24, 48, or 72 h and incubated at 37°C for 4 h. The reaction was terminated by replacing the MTT-containing medium with 150 µl dimethysulfoxide, and absorbance at 540 nm was measured on a Multiscan Mk3 spectrophotometer (Thermo Electron Corporation, USA).

### Cytotoxicity Detection

LDH cytotoxicity assays were performed according to the manufacturer's protocol (Roche, Switzerland). This colorimetric assay quantifies activity of LDH released from the cytosol of damaged cells into the supernatant and thus serves to quantify cell death [70], [71].

### Measurement of Intracellular ROS

Levels of cytosolic ROS were measured by 2′,7′-dichlorofluorescein-diacetate (DCFH-DA, Beyotime, China) as described [72]. SH-SY5Y cells were grown in a 24-well plate and incubated with ribosylated α-Syn for 8 h. Cells in the presence and absence of *D*-ribose and native α-Syn were used as controls. Cells were washed with PBS and incubated with DCFH-DA for 30 min. DCFH-DA was initially non-fluorescent and was converted by oxidation to the fluorescent molecule DCFH (λ_ex_485 nm; λ_em_538 nm). DCFH was then quantified using a CytoFluor Multi-well Plate Reader (Fluoroskan Ascent, Thermo Lab Systerms, USA).

### Flow Cytometry

Cells undergoing apoptosis were detected by double staining with annexin V-FITC/PI in the dark according to the manufacturer's instructions [73]. Cells attached to dishes were harvested with 0.25% trypsin and washed twice with cold PBS. Cell pellets were suspended in 1×binding buffer (10 mM HEPES/NaOH, pH 7.4, 140 mM NaCl, 2.5 mM CaCl_2_) at a concentration of 1×10^6^ cells/ml, and then incubated with annexin V- FITC and propidium iodide (PI) for 15 min (22–25°C) in the dark. The stained cells were immediately analyzed by flow cytometry (FAC Svantage SE, USA). Each measurement was carried out at least three times.

### Data Analysis

All values reported are means ± standard errors (SE), except where otherwise indicated. Data were analyzed with Origin 6.0 and Microsoft Excel 2003 statistical software (USA). Differences between experimental groups were considered to be significant if the probability was <0.05 in two-tailed tests. Analysis of first order rate constants in the kinetic studies were as described by Tsou [74].

## Supporting Information

Figure S1 Purification of α-Syn. A culture overexpressing α-Syn was induced with 200 µM IPTG for 3 h (lane 1, 2 and 3). After disrupting the cell by osmotic shock (lane 4), the pellet was resuspended in periplasm protein extraction buffer (lane 5). Then the supernatant was collected and purified by Q-sepharose FF (lane 6, 7) and Superdex 75 (lane 9) column. All samples were analysed by 15% SDS-PAGE.
(0.38 MB TIF)

Figure S2 N-terminal sequencing of ribosyled α-Syn digested by trypsin. The experiment conditions were the same as those in Fig. 5A. Aliquots were taken from the incubation at day 3, followed by band ‘a’ (A) and band ‘b’ (B) on gel were sequenced as described [75].
(0.15 MB PPT)
