# Supplementary figures and images for: Ribosylation Rapidly Induces α-Synuclein to Form Highly Cytotoxic Molten Globules of Advanced Glycation End Products
Source: PLoS One. 2010 Feb 4;5(2):e9052. doi: 10.1371/journal.pone.0009052 (PMC2816216; doi:10.1371/journal.pone.0009052)

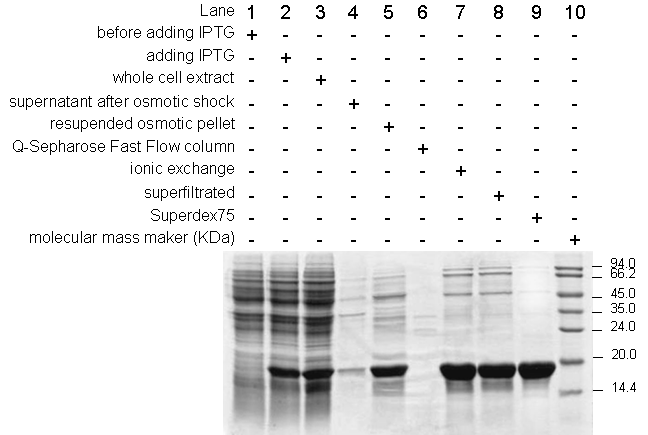

Supplement: Figure S1 — Purification of α-Syn. A culture overexpressing α-Syn was induced with 200 µM IPTG for 3 h (lane 1, 2 and 3). After disrupting the cell by osmotic shock (lane 4), the pellet was resuspended in periplasm protein extraction buffer (lane 5). Then the supernatant was collected and purified by Q-sepharose FF (lane 6, 7) and Superdex 75 (lane 9) column. All samples were analysed by 15% SDS-PAGE. (0.38 MB TIF) [file pone.0009052.s001.tif]
