# Supplementary material for: Ribosylation Rapidly Induces α-Synuclein to Form Highly Cytotoxic Molten Globules of Advanced Glycation End Products
Source: PLoS One. 2010 Feb 4;5(2):e9052. doi: 10.1371/journal.pone.0009052 (PMC2816216; doi:10.1371/journal.pone.0009052)
Supplement: Figure S2 — N-terminal sequencing of ribosyled α-Syn digested by trypsin. The experiment conditions were the same as those in Fig. 5A. Aliquots were taken from the incubation at day 3, followed by band ‘a’ (A) and band ‘b’ (B) on gel were sequenced as described [75]. (0.15 MB PPT) [file pone.0009052.s002.ppt]

## Slide 1
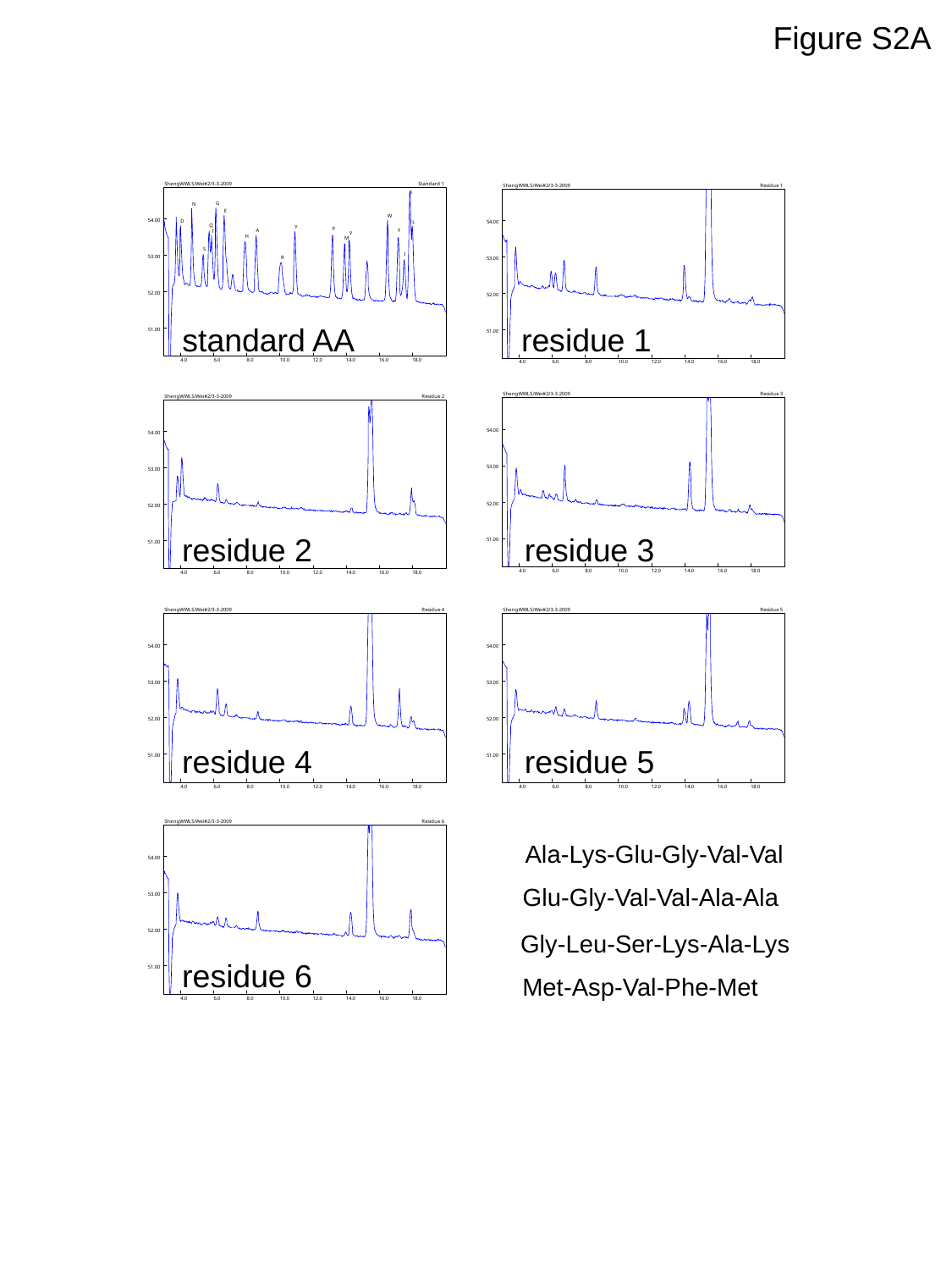

Figure S2A
standard AA
residue 1
residue 2
residue 3
residue 4
residue 5
Ala-Lys-Glu-Gly-Val-Val
Glu-Gly-Val-Val-Ala-Ala
Gly-Leu-Ser-Lys-Ala-Lys
residue 6
Met-Asp-Val-Phe-Met

## Slide 2
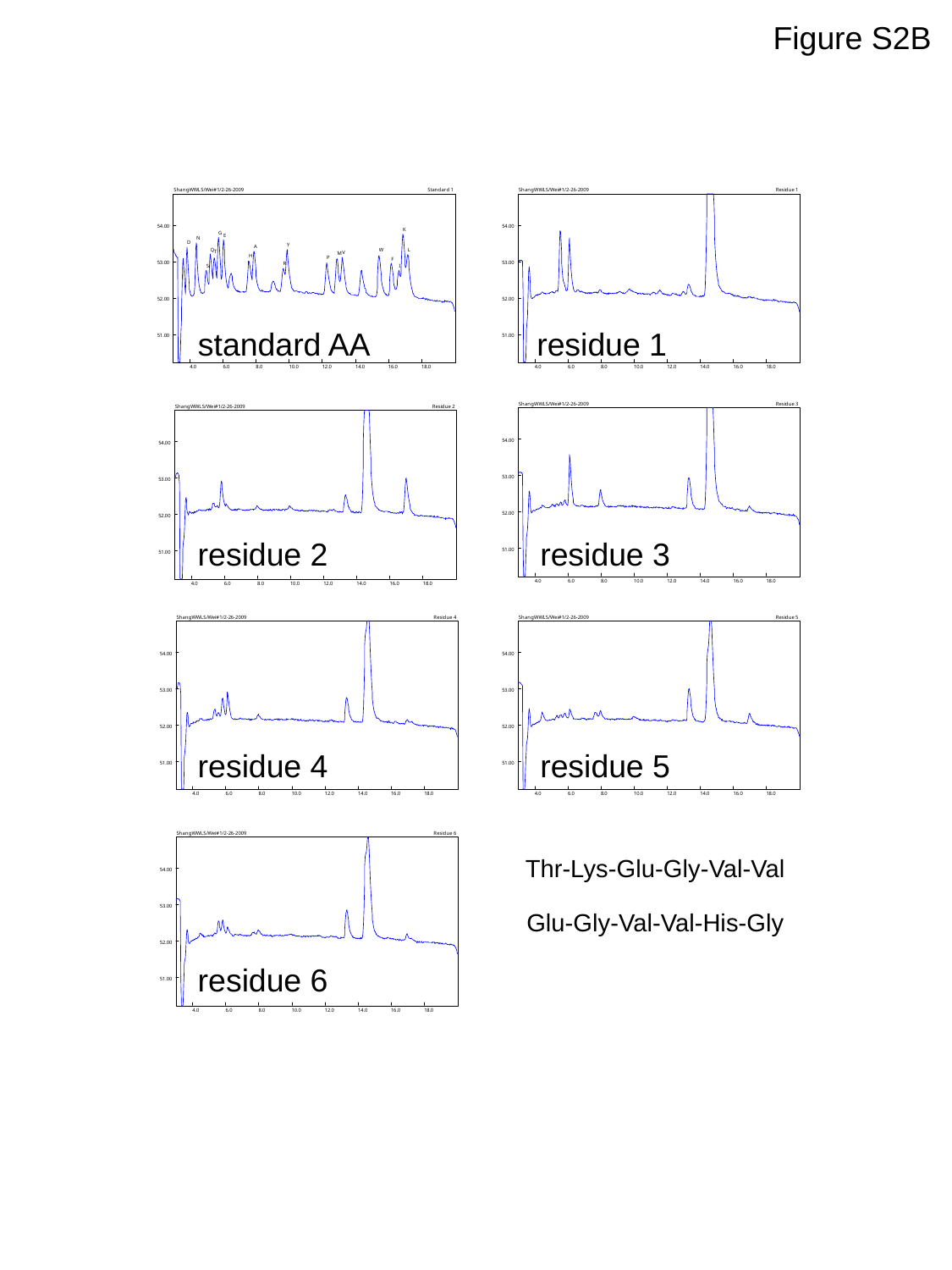

Figure S2B
standard AA
residue 1
residue 2
residue 3
residue 4
residue 5
Thr-Lys-Glu-Gly-Val-Val
Glu-Gly-Val-Val-His-Gly
residue 6
